# Heparan Sulfate Proteoglycans in Viral Infection and Treatment: A Special Focus on SARS-CoV-2

**DOI:** 10.3390/ijms22126574

**Published:** 2021-06-18

**Authors:** Valeria De Pasquale, Miriam Shasa Quiccione, Simona Tafuri, Luigi Avallone, Luigi Michele Pavone

**Affiliations:** 1 Department of Veterinary Medicine and Animal Productions, University of Naples Federico II, 80137 Naples, Italy; stafuri@unina.it (S.T.); avallone@unina.it (L.A.); 2 Department of Molecular Medicine and Medical Biotechnology, Medical School, University of Naples Federico II, 80131 Naples, Italy; m.quiccione@studenti.unina.it

**Keywords:** heparan sulfate proteoglycans, virus, SARS-CoV-2, pathogenesis, therapy

## Abstract

Heparan sulfate proteoglycans (HSPGs) encompass a group of glycoproteins composed of unbranched negatively charged heparan sulfate (HS) chains covalently attached to a core protein. The complex HSPG biosynthetic machinery generates an extraordinary structural variety of HS chains that enable them to bind a plethora of ligands, including growth factors, morphogens, cytokines, chemokines, enzymes, matrix proteins, and bacterial and viral pathogens. These interactions translate into key regulatory activity of HSPGs on a wide range of cellular processes such as receptor activation and signaling, cytoskeleton assembly, extracellular matrix remodeling, endocytosis, cell-cell crosstalk, and others. Due to their ubiquitous expression within tissues and their large functional repertoire, HSPGs are involved in many physiopathological processes; thus, they have emerged as valuable targets for the therapy of many human diseases. Among their functions, HSPGs assist many viruses in invading host cells at various steps of their life cycle. Viruses utilize HSPGs for the attachment to the host cell, internalization, intracellular trafficking, egress, and spread. Recently, HSPG involvement in the pathogenesis of SARS-CoV-2 infection has been established. Here, we summarize the current knowledge on the molecular mechanisms underlying HSPG/SARS-CoV-2 interaction and downstream effects, and we provide an overview of the HSPG-based therapeutic strategies that could be used to combat such a fearsome virus.

## 1. Heparan Sulfate Proteoglycans: Structure, Biosynthesis, Processing, and Functions

**Figure 1:**
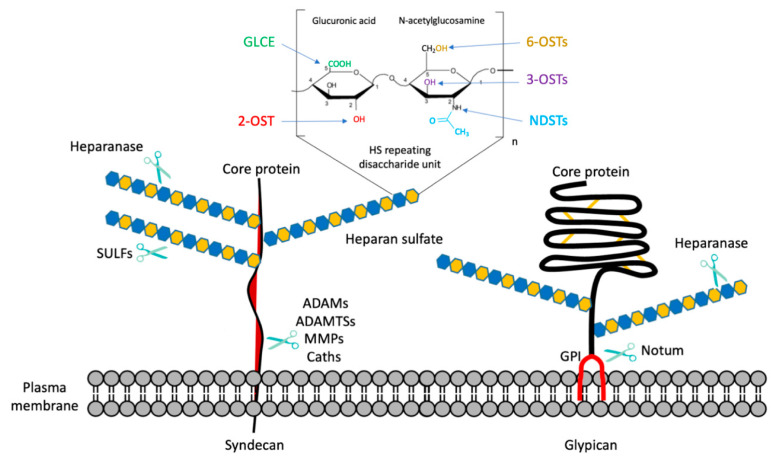
Schematic depiction of the repeating disaccharide unit in HS chains, the sites of action of biosynthetic and post-translational enzymes, and syndecan and glypican structures. NDSTs, *N*-deacetylase/N-sulfotransferases; GLCE, glucuronyl C5-epimerase; OSTs, *O*-sulfotransferases; MMPs, matrix metalloproteinases; ADAMs, a disintegrin and metalloproteinases; ADAMTSs, ADAMs with a thrombospondin motif; Caths, cathepsins; SULFs, extracellular sulfatases; GPI, glycosylphosphatidylinositol.

Proteoglycans (PGs) consist of a core protein bearing one or more carbohydrate chain of glycosaminoglycans (GAGs) [1,2]. Although structurally complex, GAG polysaccharides are simply made up of repeating disaccharide units composed of an amino sugar and one uronic acid. The uronic acid unit may be either β-d-glucuronic acid (GlcA) or its C5 epimerized form, α-l-iduronic acid (IdoA), whereas the amino sugar may consist of either glucose (Glc)-based (α-d- or β-d-glucosamine, GlcN) or galactose (Gal)-based amino sugars such as *N*-acetyl-β-d-galactosamine (GalNAc). Based on their composition, GAGs are classified as chondroitin sulfate (CS), dermatan sulfate (DS), heparin and heparan sulfate (HS), keratan sulfate (KS), and the non-sulfated hyaluronic acid (HA). In the heparan sulfate proteoglycans (HSPGs), the core protein is covalently attached to long linear HS chains composed of alternating GlcA and α-GlcN disaccharide units that can be variably *N*- and *O*-sulfated (Figure 1).

HSPG synthesis, occurring in the Golgi apparatus upon the arrival of the core protein from the endoplasmic reticulum, starts with the attachment of a tetra-saccharide linker (xylose-galactose-galactose-glucuronic acid) to a serine residue of the core protein and proceeds with the elongation of the polysaccharide backbone by the addition of GlcNAc and GlcA mediated by exostosin glycosyltransferases (EXT1/2; EXTL1/2/3). Next, the *N*-acetyl group of GlcNAc is removed and replaced by a sulfate group through the action of *N*-deacetylase/*N*-sulfotransferases (NDST1/2/3/4). The glucuronyl C5-epimerase (GLCE) promotes the epimerization of GlcA to IdoA, followed by *O*-sulfotransferase (OST) activity triggering the 2-*O*-sulfation and 3-*O*-sulfation of IdoA and GlcN, respectively, and the 6-*O*-sulfation of *N*-acetylated or *N*-sulfated GlcN residues (Figure 1). The reactions mediated by the Golgi-localized enzymes likely engage only some fraction of potentially available sugar units, giving rise to highly heterogeneous HS chains [3].

Once synthesized and exported to their localization on the cell surface and extracellular matrix (ECM), HSPGs may undergo further modifications through the action of enzymes that target either HSPG protein core or HS chains [4]. These enzymes include sheddases, the endoglycosidase heparanase, and 6-*O*-endosulfatases (Figure 1). The first type of HSPG-modifying enzymes, generically termed sheddases, target the core protein of cell surface HSPGs, triggering the release of their HS-bearing ectodomain into the extracellular milieu. The sheddases include the extracellular lipase Notum, and proteases such as matrix metalloproteinases (MMPs); ADAMs, a disintegrin and metalloproteinases; ADAMTSs, which are ADAMs with a thrombospondin motif; and cathepsins [5]. Heparanase is an endoglucuronidase that specifically cleaves HS chains, acting on the GlcA–GlcNS glycosidic bond [4,6]. As a result of the heparanase activity, shorter HS fragments are released that may either promote ECM remodeling or activate HSPG catabolism [4,7]. Extracellular endosulfatases (SULF1/2) promote 6-*O*-desulfation of HS, starting from the non-reducing end of HS chain S-domains, with a strong preference for the [Glc/IdoA(2S)-GlcNS(6S)] trisulfated disaccharides, which are mostly present within HS functional S-domains [4,8]. Hence, SULF activity has a great impact on HS binding properties and functions. The post-synthetic modifications occurring in the HSPG structure at the cell surface and ECM are cell- and tissue-specific and augment the structural heterogeneity and complexity of HSPGs, features that account for their wide range of functions [4,9].

The main classes of HSPGs include: (a) cell-surface-localized syndecans, characterized by an extracellular domain harboring HS chains, a single transmembrane domain, and a short C-terminal cytoplasmic domain; (b) glypicans, attached to the cell membrane via a glycosylphosphatidylinositol (GPI) anchor, bearing HS chains near the juxtamembrane region (Figure 1); (c) perlecan, agrin, and collagen type XVIII localized at the ECM, including the basement membrane zone; and (d) the intracellular proteoglycan serglycin, which may vary in its GAG composition depending on the cell type.

Ubiquitously expressed on the cell surface and ECM of all cell types, HSPGs regulate numerous signaling pathways involved in developmental and physiopathological processes [10,11,12,13,14,15,16,17]. Beside an important structural role, HSPGs—through either their negatively charged sulfated moieties of HS chains or the protein cores—interact with a variety of ligands regulating their distribution, availability, and signaling activity. Hence, HSPGs play fundamental roles in cell proliferation, differentiation, adhesion, migration, survival, autophagy, inflammation, immune defense, and many other cellular processes [11,12,13,14,15,16,17,18,19,20]. HSPGs serve as co-receptors for morphogens and growth factors, allowing a proper presentation to their cognate receptors, either in cis (on the same cell) or in trans (on adjacent cells), thus facilitating receptor activation and downstream signaling [1,2,3,4,9,14,15,18,19,20]. In some cases, HSPGs may also act as receptors themselves [1,2,3,9,10,18,19,20]. In addition, HSPGs play a crucial role in endocytosis and vesicular trafficking, thus regulating the movement of molecules between intracellular and extracellular compartments [1,2,21,22,23]. In particular, HSPGs promote the internalization of a variety of macromolecules such as cationic polymers, liposomes, DNA, RNases, cancer cell exosomes, cell-penetrating peptides, protein aggregates, and pathogens [1,2,3,9,10,21,22,23,24]. Among pathogens, many viruses hijack HSPGs to enter and to infect host cells, although with different mechanisms [24,25].

## 2. Molecular Mechanisms by Which Viruses Exploit Heparan Sulfate Proteoglycans to Infect Host Cells

Some viruses take advantage of the electrostatic interactions between the negatively charged sulfated HS chains and the basic residues of their surface or capsid proteins to increase their concentration at the host cell surface, thus enhancing their binding to specific entry receptors [24,25]. Table 1 lists the viruses whose infection in the human organism is strictly dependent on their ability to bind the cell surface HSPGs [26,27,28,29,30,31,32,33,34,35,36,37,38,39,40,41,42,43,44,45,46,47,48,49,50,51,52,53,54,55,56,57,58,59,60,61,62,63,64,65,66,67,68,69].

The interaction of the viral gp120 envelope protein with HS, prior to CD4 receptor recognition, increases the infectivity of the human immunodeficiency virus (HIV) by pre-concentrating the virion particles at the cell surface [37,38,39]. Hepatitis C virus (HCV) hijacks apolipoprotein E (apoE) to interact with HS structures, prior to sequential interactions with cellular entry factors such as the scavenger receptor SRB1, the tetraspanin CD81, and two tight junction proteins, claudin-1 and occluding [44,45,46,47]. In particular, syndecan-1 and syndecan-4 serve as major cellular factors for HCV attachment to hepatocytes [45,46]. The binding of the major capsid protein, pORF2, to the HSPGs, specifically syndecans, leads to hepatitis E virus (HEV) enrichment on the cell surface, allowing subsequent interaction with entry receptors [49,50]. The respiratory pathogen human metapneumovirus (HMPV) uses HSPGs to bind to target cells and undergoes clathrin-mediated endocytosis and membrane fusion in endosomes. The binding of the HMPV fusion protein F with cell surface HSPGs is mandatory for infection [57,58]. The cell surface HS functions as the first attachment host factor for rabies virus (RABV) through its binding to the viral glycoprotein (G), thus supporting subsequent viral interaction with entry receptors including the nicotinic acetylcholine receptor, the neuronal cell adhesion molecule (NCAM), and the nerve growth factor receptor p75NTR [59,61].

In some cases, virus attachment to HSPGs allows conformational changes of the viral proteins involved in the entry, facilitating their interaction with uptake receptors and subsequent infectious internalization. Several human papilloma virus (HPV) serotypes depend on HSPGs for their initial attachment to the host cells [51,52,53], and it has been shown that, following the interaction with HSPGs, the HPV capsid proteins L1 and L2 undergo conformational changes mediated by cyclophilin B, kallikrein-8, and furin, resulting in reduced affinity for HSPG binding, and transfer to entry receptors such as α6 integrins, epidermal growth factor receptor (EGFR), and tetraspanins [54]. Among the viruses whose interaction with HSPGs on the host cell surface is a prerequisite for entering and infecting target cells, there are many types of coronaviruses, including human coronavirus NL63 (HCoV-NL63) and severe acute respiratory syndrome coronavirus-2 (SARS-CoV-2) [27,28,66]. Both these viruses employ the functional angiotensin-converting enzyme 2 (ACE2) receptor to enter and infect the host cells; however, the first attachment of HCoV-NL63 involves the viral membrane (M) protein binding to HSPGs [28], whereas SARS-CoV-2 uses the spike (S) protein to interact with HSPGs [66]. The interaction of the S protein of SARS-CoV-2 with the cell surface HSPGs triggers a conformational change of the receptor-binding domain (RBD) of the S protein that favors the binding of the virus to its specific receptor (ACE) [66].

At times, HSPGs may serve as viral receptors themselves [24,36,55,62,70,71,72,73]. This is the case for herpes simplex virus serotypes 1 and 2 (HSV-1 and HSV-2), whose viral envelope glycoproteins gB and gC bind HSPGs to promote attachment, to slide down membrane projections such as filopodia, and to reach the cell body for membrane penetration [73]. Then the binding of gD to nectin-1 or 3-*O*-sulfated HS triggers conformational changes recruiting gB, gH, and gL for membrane fusion leading to capsid release in the cytoplasm [36]. Similarly, the human respiratory syncytial virus (RSV), which is characterized by three envelope proteins—the attachment glycoprotein (G), the fusion protein (F), and the small hydrophobic protein (SH)—uses its G and F proteins to interact directly with HSPGs and to infect host cells [62]. The subsequent fusion process mediated by the F protein allows entry of the viral genome into host cells. The host cell HSPGs also serve as initial attachment receptors for the Merkel cell polyomavirus (MCV) prior to secondary interactions with a sialylated co-factor during the infectious entry process [55].

Many viruses exploit HSPG-mediated endocytosis to enter host cells [74]. The HSPG-regulated endocytic pathways utilized by the virus to enter host cells include clathrin-mediated uptake, or caveolae/cholesterol-dependent endocytosis, and macropinocytosis. Investigations on the human hepatitis B (HBV) entry pathway have demonstrated that the cellular uptake of the virus is driven by HSPG-mediated endocytosis rather than by the cell-surface sodium taurocholate cotransporting polypeptide (NTCP) receptor [75]. The proposed model implies that the L protein domains of HBV are involved in the attachment to HSPGs on human hepatic cells, the initiation of endocytosis, the interaction with NTCP in endosomes triggering membrane fusion, and the subsequent endosomal escape. Interestingly, a targeted RNA interference entry screen allowed researchers to identify glypican-5 as a preferential HBV entry factor; because this HSPG is highly expressed on the liver, the HBV-glypican-5 interaction may partly account for the strong hepatotropism of HBV [42]. Furthermore, a model for HPV entry into host cells suggests that the HPV endocytosis occurs after the binding of capsid proteins to HSPGs on either the epithelial cell surface or the basement membrane, and other signaling molecules such as growth factors and α6-integrins [51]. On the other hand, in HIV infection, HSPGs interact with both gp120 glycoprotein and the trans-activator of transcription (Tat) protein, which enhances transcription and viral virulence during infectivity and promotes virus internalization into a variety of different cell types through caveolar endocytosis [76,77]. Finally, HSPGs have been suggested to serve as assisting cofactors for ACE2-mediated endocytosis of HCoV-NL63 [27] and SARS-CoV-2 [78].

Some viruses that do not require binding to HSPGs to attach and to infect host cells may acquire HSPG dependence following intra-host or cell culture adaptation. There is abundant evidence that several viruses—including rhinoviruses [79,80], Coxsackie virus B3 [81], Sindbis virus [82,83], Ross River alphavirus [84], flavivirus tick-borne encephalitis virus [85,86], and others—during repeated passage in cell culture undergo adaptation changes leading to an augmented ability to binding HS, a phenomenon that may provide a selective advantage to the viruses [25]. Similar viral adaptations occurring in cell cultures may also take place during human infections, generating viral variants that can show different tropism, virulence, and pathogenicity than parental viruses [25,87,88,89,90,91]. One example is provided by enterovirus 71 (EV71), whose mutation acquired during the infection of an immunocompromised host enabled the virus to bind HS, thus modifying viral tropism in neural, intestinal, and respiratory tissues [87]. On the other hand, mutants of JC polyomavirus, the causative agent of progressive multifocal leukoencephalopathy, show an increased ability to bind HSPGs and infect neural cells, which express high levels of syndecans and glypicans [25,88]. Several SARS-CoV-2 mutations affecting the S protein sequence have emerged [89,90,91], but additional studies are needed to establish whether and how such mutations impact the ability of the S protein to bind HSPGs and, in turn, on infection, tropism, immunity, and pathogenesis of SARS-CoV-2.

Of note, cell culture adaption mediated by HS may promote attenuation of viral virulence. Indeed, in some cases, HS attachment may inhibit rather than enhance the dissemination of HS-binding viruses [32,92,93,94,95,96,97,98]. A trapping effect of cell surface HSPGs has been demonstrated in vivo through the injection into mice of two enterovirus A71 mutants that resulted in a higher virulence of the HS-non-binding mutant with respect to that of the HS-binding one. Indeed, although HSPGs are expressed by many cultured cell lines and increase the infection by a subset of EV-A71 strains, they are not expressed by cells that express the SCARB2 viral receptor at high levels in vivo. Thus, HS-positive cells merely adsorb the virus and do not contribute to replication or dissemination of the virus in vivo [92]. Attenuation of viral pathogenicity through the acquisition of HS-binding ability in cell cultures has also been reported for DENV [93], Sindbis virus [83], encephalitis viruses [94], foot and mouth disease virus [95], and others. On the other hand, in vivo HSPGs may not only facilitate the concentration of the virus at the cell surface, enhancing the probability of access to the related entry receptors, but they may also trap the viral particles at the surface of non-permissive cells and mediate in trans infection by allowing the virus to interact with entry receptors on permissive cells [96,97]. For example, the high levels of syndecan-3 expressed by the endothelial cells of lymphoid tissues capture HIV particles and present them to permissive T cells [98].

## 3. Role of Heparan Sulfate Proteoglycan Biosynthetic and/or Modifying Enzymes in Viral Infections

Beyond the evidence that viruses use HSPGs for attachment and entry into host cells, either HSPG biosynthetic or post-synthetic modifying enzymes have emerged as critical players in the viral invasion of target cells at various steps of their life cycles [4,7,99]. Table 2 reports a list of the viruses whose ability to enter and to infect host organisms is regulated by the differential activity of HSPG biosynthetic and/or modifying enzymes [100,101,102,103,104,105,106,107,108,109]. The 3-*O*-sulfation of HS chains catalyzed by 3-*O*-sulfotransferases (3-OSTs) is required for the internalization and spread of human cytomegalovirus (CMV) [100] and HSV-1 [33,70,72], whereas 3-OST isoform B is downregulated in the hepatocytes of chronic HBV infection [106]. Furthermore, while HS modified by the 3-OST isoform 3 has been suggested to increase SARS-CoV-2 cell-to-cell fusion [109], a preferential recognition of the S protein receptor binding domain (RBD) by *N*- and 6-*O*-sulfated HS sequences has been identified [66]. In addition, 6-*O*- and *N*-sulfation of GlcNAc of HS is a critical determinant for coxsackievirus B3 variant PD interaction with the host cell [101] as well as for HCV infection [44,47], and efficient rabies virus infection of target cells [59], whereas *N*-sulfation, but not C6-*O*- or C2-*O*-sulfation, is important for RSV infection [63,108]. Furthermore, 6-*O*-sulfated groups of HS are essential to promote the interaction of HIV glycoprotein gp120 with HSPGs on the surface of host cells, enabling virus attachment, fusion, and entry into the cells [37,38]. A similar requirement for 6-*O*-sulfation has also been shown for the interaction of HEV pORF2 capsid protein with the syndecans on the cell surface [49].

A key role of HSPG-modifying enzymes such as sheddases, heparanases, and endosulfatases (SULFs) in the pathogenesis of viral infections has been demonstrated [7,99]. During the infection process, HSPGs can trap the viral progenies, inhibiting their release and spread; hence, HS-binding viruses have developed mechanisms to circumvent such a problem. These mechanisms may involve either the upregulation of heparanase that degrades the HS polysaccharide backbone or the activation of proteases that shed the protein core of HSPGs [99]. Upregulation of heparanase in response to HSV-1 infection results in the shedding of HS chains from the plasma membrane of infected cells that lose the capability to trap the newly synthesized virions, thus allowing their spread to other cells and tissues [104,105]. Syndecan-1 shedding by heparanase is an essential step in the dengue virus (DENV) infection; this action leads to hyperpermeability of human endothelial cells in vitro and systemic vascular leakage in vivo [102,103]. These effects are mediated by the secreted DENV non-structural protein 1 (NS1), which disrupts the endothelial glycocalyx layer through the activation of sialic acid degradation and HSPG shedding. In particular, NS1 upregulates sialidases and heparanase and activates cathepsin L, which in turn activates heparanase by enzymatic cleavage [102]. Inhibition of syndecan-1 shedding by MMPs, ADAMs, and/or heparanase greatly reduces cellular uptake of HPV serotype 16 and subsequent infection [7,53,107]. During infection, HPV-16 mainly attaches to ECM components of keratinocytes through HS chains of syndecan-1, and the action of HSPG processing enzymes is relevant to the release of infectious viral particles from the ECM and to an efficient infection of keratinocytes [53]. The involvement of heparanase, acting by lowering *N*-sulfation and iduronic acids units of HS chains, thus reducing infection, has been shown in different cell lines infected by RSV [63,108]. A contribution of heparanase to the pathogenesis of SARS-CoV-2 infections has been also suggested because elevated activity of the enzyme together with high levels of HS were found in the plasma of patients affected by SARS-CoV-2 infectious disease (COVID-19); these factors are associated with the severity of the disease. The proposed mechanisms to explain heparanase involvement in the severe forms and worsened outcomes of COVID-19 include its well-established roles in the degradation of the endothelial glycocalyx and the activation of inflammatory responses [110]. These findings and other evidence suggest that biosynthetic and/or post-translational modifying enzymes are important for the interaction of viruses with host cells and the infection process. However, further structure–function analyses of modified-HS chains in different tissues and organs might provide more insights into the pathogenesis of viral diseases and could be useful for developing novel potential diagnostic tools and therapeutic interventions.

## 4. Heparan Sulfate Proteoglycan Involvement in the Pathogenicity of SARS-CoV-2

SARS-CoV-2 is a member of the *Coronaviridae* family, the order *Nidovirales*, and the former genus *Coronavirus*, which has been split into four genera (see below). Coronaviruses (CoVs) are enveloped positive single-stranded RNA viruses with a large genome of 28–32 kb that infect a wide spectrum of animal species including humans [111]. They are classified into four genera: *Alphacoronavirus*, *Betacoronavirus*, *Gammacoronavirus*, and *Deltacoronavirus*. However, only seven types of CoVs belonging to the *Alphacoronavirus* and *Betacoronavirus* genera cause infections in humans, with variable outcomes. Four of them, namely HCoV-229E, HCoV-OC43, HCoV-NL63, and HCoV-HKU1, cause mild and self-limiting infections of the upper respiratory tract, whereas the SARS-CoV, the recent SARS-CoV-2, and the Middle East respiratory syndrome coronavirus (MERS-CoV) result in serious respiratory tract infections leading to pneumonia and renal failure, with an elevated mortality rate [112].

The coronavirus particles are composed of a nucleocapsid protein (N) surrounding the viral genomic RNA, and an envelope bearing a membrane protein (M), an envelope protein (E), and a spike protein (S) [112,113]. However, additional structural proteins have been identified for some CoVs, such as hemagglutinin esterase and accessory open reading frame (ORF) proteins that are not essential for viral replication but seem to have a role in viral pathogenesis [114]. Although viral entry into host cells may involve different structural proteins, the major envelope S protein mediates the attachment of CoV particles to cell surface molecules and receptors as well as the fusion between the virus and the cell membrane. The S protein contains three segments: a large ectodomain, a transmembrane anchor, and a short tail. The ectodomain is composed of two subunits: the S1 subunit is involved in the binding of receptors on the host cell surface, and the S2 subunit is required for fusing host and viral membranes. In most CoVs, the C-terminus of the S1 (S1-CTD) domain contains one or more receptor binding sites (RBSs), which may be highly divergent among different CoV types, whereas the N-terminus of the S1 region (S1-NTD) is more conserved and mainly contributes to the initial binding to the attachment factors on the host cell surface [113]. Among the receptors recognized by the S1-CTD are the zinc aminopeptidase N (APN) for HCoV-229E [115]; the serine dipeptidyl peptidase 4 (DPP4) for MERS-CoV [116]; and the zinc peptidase ACE2 for HCoV-NL63, SARS-CoV, and SARS-CoV-2 [26,66,117]. However, in SARS-CoV-2, the cleavage of S protein by host furin into the S1 and S2 subunits generates a polybasic sequence in S1 that binds the cell surface neuropilin-1 (NRP1) receptor, potentiating virus infectivity [67,68]. Although both SARS-CoV and SARS-CoV-2 use the ACE2 receptor to infect host cells, the SARS-CoV-2 S1 protein binding to NRP1 could explain the different tropism of the two related viruses, with SARS-CoV infection occurring predominantly in the lower respiratory system [118] whereas SARS-CoV-2 rapidly spreads through active pharyngeal viral shedding [119]. Indeed, while ACE2 is expressed at low levels in respiratory and olfactory epithelial cells [120], NRP1 is highly expressed in the respiratory and olfactory epithelium. Recently, a novel receptor, the tyrosine-protein kinase receptor UFO (AXL) that specifically interacts with the N-terminal domain of SARS-CoV-2 S protein, has been shown to promote the entry of SARS-CoV-2 into the cells of the respiratory system, such as pulmonary and bronchial epithelial cells [69].

Prior to the specific interaction with the entry receptors, CoV infection requires the initial binding of the viral envelope proteins with host cell surface molecules such as carbohydrates and glycoproteins that leads to the local viral enrichment prior to internalization. For example, HCoV-HKU1 and HCoV-OC43 bind *O*-acetylated sialic acid [121], although HCoV-HKU1 also recognizes the major histocompatibility complex class I C (HLA-C) as attachment molecule [122], and HCoV-OC43 has also been shown to attach to HS during adaption in cell culture [123]. Similarly, binding of SARS-CoV proteins to dendritic cell-specific intercellular adhesion molecule-3-grabbing nonintegrin (DC-SIGN) and DC-SIGN-related protein (also termed L-SIGN) enhances infection [124]. MERS-CoV exploits carcinoembryonic antigen-related cell adhesion molecule 5 (CEACAM5) [125], tetraspanin CD9 [126], and the 78-kDa glucose-regulated protein (GRP78) [127] as attachment factors that facilitate viral entry. On the other hand, effective adhesion to cell surface HSPGs enhancing the infection process has been reported for different types of animal and human CoVs, including CoV-NL63, SARS-CoV, and SARS-CoV-2 [27,66,81]. In particular, HS has been definitively identified as an attachment receptor for SARS-CoV-2 infection in in vitro human lung epithelial cells and ex vivo human lung tissue explants [128].

Since 2020, a substantial number of studies have addressed the role and the mechanisms underlying the interaction between the viral envelope S protein of SARS-CoV-2 and the HSPGs that provide the first anchoring sites for the virus on the host cell surface [66,81,129,130,131,132]. It has been established that the binding of the S protein of SARS-CoV-2 to the host cells requires the engagement of both HS and ACE2 [131], thus suggesting that HSPGs act as a co-receptor for the S protein interaction with the ACE2 entry receptor [66]. The binding of S1-NTD to HSPGs allows the initial contact between SARS-CoV-2 and the host cell, facilitating the concentration of the virus at the cell surface and its access to ACE2. In particular, the ectodomain of the S protein interacts with cell surface HS chains through the S1 receptor-binding domain (RBD) that binds the peptidase domain of ACE2 [133]. Indeed, the trimeric S protein of SARS-CoV-2 contains a group of positively charged amino acid residues such as R346, R355, K444, R466, and R509 that are localized in a position adjacent to the ACE-binding site and exposed in the RBD, thus representing the potential sites of interaction with HS chains [66]. Interestingly, a single RBD may simultaneously bind both cell surface HS chains and the ACE receptor [66]. Surface plasmon resonance (SPR) and circular dichroism spectroscopy have demonstrated that heparin, which is structurally similar to HS, binds and induces a conformational change in the RBD domain of the SARS-CoV-2 S1 protein [134]. This binding is more dependent on the presence of 2-*O*- or 6-*O*-sulfated groups than *N*-sulfated HS domains. Furthermore, studies support a model in which the RBD of SARS-CoV-2 S protein lends sequence specificity for HS on target cells, while an additional binding site at the S1/S2 proteolytic cleavage site enhances the affinity of the binding to HS [135,136].

The elegant work by Clausen and co-workers provides strong data supporting a model in which HSPGs serve as “collectors” of viral particles and “mediators” of the RBD-ACE2 interaction, thus triggering more efficient infection [66]. After the contact of the SARS-CoV-2 S protein with HSPGs and ACE2 receptor, host cell membrane proteases prime the S protein to carry out efficient internalization through the process of membrane enfolding [137]. In particular, the S2 subunit of the S protein ectodomain drives the fusion of the viral envelope with the membrane of host cells. However, virus-cell membrane fusion requires S protein cleavage at the S1/S2 boundary site by furin. The furin cleavage of the S protein is essential for efficient replication of SARS-CoV-2 in human lung epithelial cells [135]. Furthermore, the S2 subunit undergoes an additional cleavage into fusion peptide (FP) and S2′ domains through the action of the cellular serine protease TMPRSS2 (transmembrane protease serine-2) that cleaves the S2’ site, or the endosomal cysteine proteases cathepsin B and L (CatB/L) [136,137]. The activity of cysteine cathepsins is strictly regulated by HSPGs that promote their autocatalytic activation as well as conformational changes that increase their affinity for the substrate and enhance their activity [5,138].

Some reports suggest that the HSPGs also act as assisting cofactors in SARS-CoV-2 endocytosis. Through this route, the ACE2-bound virus may use the endosomal pathway to move through the cytoplasm, where it starts replication and exits from the cells to transfect the neighboring cells [81]. The contribution of HSPGs to this process has been suggested based on evidence showing that SARS-CoV-2 entry into the cells is prevented by ablating genes involved in HSPG biosynthetic machinery or employing HS mimetics targeting endocytic pathways [81,139]. Furthermore, a Food and Drug Administration (FDA)-approved drug that blocks HS-dependent endocytosis of α-synuclein (α-Syn) fibrils is able to inhibit SARS-CoV-2 infection [22,140]. Finally, in vitro cellular assays supported by computational studies showed that HSPGs modified by the 3-OST isoform 3, but not the 3-OST isoform 5, increase the S-protein-mediated cell-to-cell fusion of SARS-CoV-2, thus suggesting a role for HSPGs in viral spread [109].

The primary site of SARS-CoV-2 infection is the human upper and lower respiratory tract [141,142], although the virus can infect other organs [143,144,145]. Once SARS-CoV-2 enters the host through the respiratory tract, its first targets are the airway and alveolar epithelial cells, the vascular endothelial cells, and the alveolar macrophages [119]. HSPGs play important roles in maintaining parenchymal architecture and pulmonary homeostasis and facilitating cell signaling required for lung development and functions [146]. While it is unclear whether alveolar epithelial cell surface HS contributes to the epithelial surface layer, the sulfation pattern of epithelial HSPGs significantly impacts the alveolar intercellular signaling and the epithelial cell phenotype [147,148]. The HSPGs abundantly expressed on the alveolar basement membrane serve to connect the alveolar endothelium and epithelium, whereas in the pulmonary endothelial glycocalyx they contribute to the endothelial barrier function [149]. In pulmonary vasculature, HSPGs play an important role in angiogenesis and smooth muscle cell activation [146].

Abundant evidence has shown that SARS-CoV-2 infection promotes endothelial dysfunction and vascular leakage [150,151,152,153]. Autopsies on patients who died of COVID-19 have revealed in the lungs severe endothelial injury associated with the presence of intracellular virus and disrupted cell membranes, and widespread vascular thrombosis with microangiopathy [154,155]. Viral inclusions into endothelial cells of glomerular capillary loops and widespread endotheliitis in the lung, heart, liver, kidney, and gut were detected in the autoptic specimens of patients affected by severe forms of COVID-19 [152]. The multi-organ endothelial dysfunctions observed in COVID-19 patients are likely due to the ubiquitous expression of SARS-CoV-2 receptor ACE2 in organs such as the lung, gut, kidney, brain, testis, heart, and mainly in the vascular system, where high levels of the receptor are present in the endothelial cells of either small or large arteries and veins [156,157]. However, the expression levels of ACE2 in the tissues (including the vascular system) of males and females as well as of younger and older people do not fully explain the different severity of COVID-19 observed in such distinct populations. Hence, other host cell factors likely play a determinant role in the development of lethal complications in COVID-19 patients.

Under the condition of old age and/or comorbidities, including chronic obstructive pulmonary disease, hypertension, cardiovascular disease, diabetes, and obesity, damage of the vascular endothelial glycocalyx has been associated with poor prognosis in severe COVID-19 patients [158]. The glycocalyx is a gel-like layer covering the surface of all living cells; it is composed of a membrane-binding domain consisting of sialic acid-containing glycoproteins, syndecans, HS, and hyaluronic acid (HA). The endothelial glycocalyx plays a critical role in maintaining vascular homeostasis and regulating the interaction between vascular endothelial cells and blood components [159,160]. A variety of cellular stresses may damage the vascular endothelial glycocalyx, and the damage is known to be sex specific, mostly observed in men [161]. Systemic degradation of the vascular endothelial glycocalyx occurring in serious infections and other severe pathologies such as sepsis and inflammation, atherosclerosis, ischemia and hypoxia, diabetes, and renal diseases leads to thinning of the glycocalyx layer and increased vascular permeability [158,159,160,161,162,163]. Elevated concentrations of fragmented vascular endothelial glycocalyx, such as syndecan-1, syndecan-4, HA, and HS, have been observed in the blood of patients affected by chronic kidney disease [164], acute decompensated heart failure [165], diabetes [166], cardiac surgery [167], Crohn’s disease [168], and others. Elevated levels of HS were found in the plasma from subjects with respiratory failure due to lung injury, and HS concentrations correlated with intensive care unit length of stay [169]. Recently, circulating levels of fragmented vascular endothelial glycocalyx have been detected in sublingual capillaries of patients with COVID-19 [158]. Damage of the vascular endothelial glycocalyx occurs more easily in elderly people than in young people, and in people with common comorbidities [158]; researchers suggest that this difference represents a mechanism for the development of fatal complications in COVID-19 patients. Furthermore, sex differences in COVID-19 severity and mortality could derive from sex differences in the vascular endothelial glycocalyx constituents, including HSPGs [158,161]. Thus, as a consequence of the strict requirement of the S protein binding to HS for ACE interaction, and the prominent role of HSPGs in the structure and function of the vascular endothelial glycocalyx, it follows that the extraordinary structural variety of HSPGs generated by synthetic and post-synthetic modifying enzymes as well as the key regulatory functions exerted by HSPGs in different cell types and tissues—which depend on the sex and age of individuals [170,171]—might strongly contribute to the tissue tropism [141,142,143,144,145,146], as well as the different susceptibility of distinct patient populations to SARS-CoV-2 infection [158,161,172,173].

## 5. Perspective for Therapeutic Intervention against SARS-CoV-2 by Targeting HSPGs

**Figure 2:**
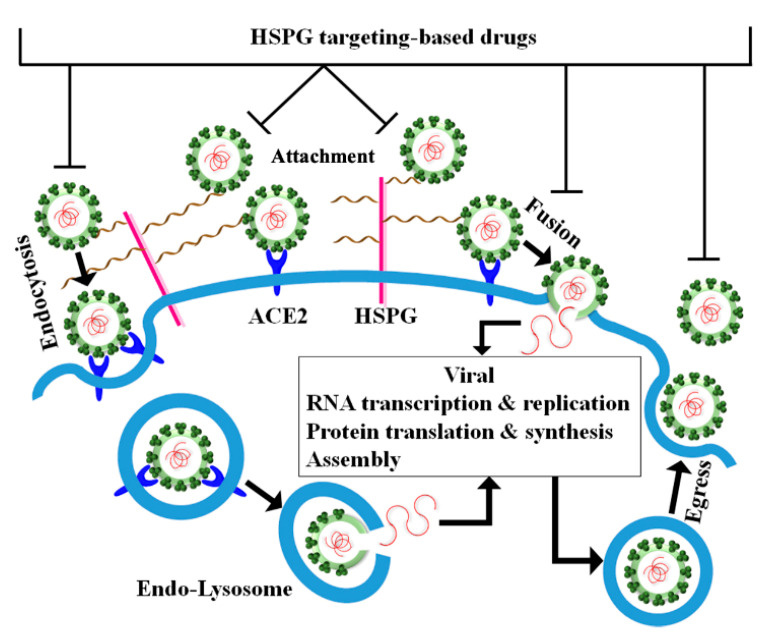
Schematics of the potential action of HSPG-targeting drugs in SARS-CoV-2 infection. HS binding compounds may compete with the viral particles for attachment to the HSPGs, thus inhibiting the viral engagement of HS chains and the subsequent access to the ACE2 receptor. Consequently, the viral entry by either fusion or endocytosis may be impaired.

The growing evidence on the involvement of HSPGs in the SARS-CoV-2 pathogenicity has prompted many researchers to suggest the development of therapeutic strategies targeting HSPGs to combat the infection and transmission of the virus in the human population. Drugs targeting HSPGs represent valuable candidates to interfere with the viral attachment to target cells, the early stages of virus–receptor interaction, the virus-cell membrane fusion, the viral endocytosis, and the viral spread (Figure 2).

For this purpose, the development of antibodies directed against HS, heparin/HS-based oligosaccharides, small HS mimetics, HS-degrading lyases, inhibitors of the HS biosynthetic machinery, and inhibitors of serine or cysteine proteases (i.e., cathepsins) involved in the viral endocytosis might prove useful therapeutic strategies to fight SARS-CoV-2 infection [9,20,38,66,75,81,132,139,174,175,176,177,178]. Table 3 reports some examples of HSPG-targeting therapeutics that have been proposed to be used alone or in combination with other drugs to block the infectious cycle of SARS-CoV-2.

The therapeutics listed in Table 3 are FDA-approved drugs for the treatment of diseases other than SARS-CoV-2 infection, although an antiviral activity has been established for most of them [179,180,181,182,183,184,185,186,187,188,189,190,191,192,193,194,195,196,197,198,199,200,201,202,203,204,205,206,207,208,209,210].

Unfractionated (full length) and low-molecular-weight heparins are currently used for treating SARS-Cov-2-associated coagulopathy and thrombosis that contribute to the morbidity and mortality of the disease [154,211]. However, based on the co-receptor activity exerted by cell surface HS (the GAG class of which heparin is composed) in the entry and infectivity of SARS-CoV-2 discussed in the previous section, beyond anticoagulation, alternative beneficial mechanisms of action for heparin in patients with COVID-19 including direct SARS-CoV-2 antiviral activity have been proposed [179,180,181,182,183]. Heparin could serve as a competitive inhibitor for viral entry, thus reducing infectivity. The evidence that shorter heparins do not significantly bind S protein of SARS-CoV-2 [134] strongly suggests that the beneficial effects of unfractionated heparin could be due not only to its anticoagulant activity, but also to an antiviral action [130]. The antiviral activity of heparin against SARS-CoV-2 has been recently demonstrated in Vero E6 cells inoculated with a Dutch SARS-CoV-2 isolate [181]. In this study, heparin prevented SARS-CoV-2 infection and subsequent replication with a half-maximal inhibitory concentration (IC_50_) below 125 μg/mL. Although to date no clinical data linking heparin therapy to antiviral outcomes exist for COVID-19 patients, the use of heparin and other HS derivatives has the potential for future clinical applications [182,183]. The potential risks and off-target effects of heparin therapy, besides significant bleeding, still need to be identified due to the heterogeneous structure of unfractionated heparin. Heparin is formed by a mixture of distinct biologically derived HS chains that contain not only the pentasaccharide sequence necessary for antithrombin 3 activation (and thus anticoagulation), but also a wide variety of other non-anticoagulant sulfated sequences which allow heparin binding to several growth factors, potentially promoting both organ-protective [212,213] and organ-harmful [214,215] effects.

Heparin-binding peptides (HBPs) are non-anticoagulant natural or synthetic peptides able to antagonize the attachment of viruses to HSPGs [175]. Some of these peptides show antiviral activity toward human CMV, HSV-1, and HSV-2 [184,185]. Peptide-based therapeutics such as HBPs enter the cells through an endocytic pathway similar to that of viruses, but, unlike viruses, they cannot escape the endosomal/lysosomal system, and thus are sorted to lysosomes for degradation; this ability enables them to prevent viral egress and cell-to-cell spread of infection. To overcome this limitation, HBPs have been conjugated with a CRM-197 carrier protein, a nontoxic mutant of diphtheria toxin, which itself is an HBP with four heparin-binding domains [186], and it is able to escape the endosomal pathway to enter cytoplasm [187]. The HBP-CRM-197 conjugates enter the cells and by escaping the endosomal pathway bind cytoplasmic viruses, thus inhibiting viral replication and/or cell-to-cell transmission. Because an HBP-CRM-197 conjugate is already in a clinical trial for the treatment of systemic amyloidosis [188,189], it has been proposed as a potential therapeutic option for COVID-19 [175].

A high-throughput drug screen for inhibitors that block the SARS-CoV-2 entry pathway coupled to biochemical and mass spectrometry analyses resulted in the identification of two classes of drugs categorized based on their ability to bind heparin: BNTX, sunitinib, and tilorone with no affinity to heparin, and mitoxantrone with high affinity to heparin [81]. Among these drugs, tilorone and mitoxantrone are already used as broad-spectrum antiviral agents [197,205,210]. Of note, recent evidence demonstrated that when added to Vero cells prior to viral infection, tilorone exhibits antiviral activity against SARS-CoV-2 infection with an IC_50_ value of 4.09 µM [139]. Lactoferrin, an iron-binding protein of the ferritin family secreted by glandular cells and present in most body fluids, is also considered a broad-spectrum antiviral agent, and it has been proposed as a potential preventive and adjunct treatment for COVID-19 [190,191,192]. The antiviral mechanisms of lactoferrin are based on its ability to bind either HSPGs on the host cell surface and, consequently, to reduce viral internalization into host cells [193,194], or to bind the viral particles to divert them from target cells [195]. Lactoferrin has shown antiviral activity in cell culture against several human coronaviruses, including CoV-OC43, CoV-229E, CoV-NL63, SARS-CoV, as well as SARS-CoV-2 [192], thus suggesting its potential use in SARS-CoV-2 infection. In addition to its oral bioavailability, the lack of immunogenicity, and the broad-spectrum antiviral activity, lactoferrin has also shown anti-inflammatory and immunomodulatory activities in severe viral infections [196], thus resulting in a promising drug candidate for COVID-19.

Among HS mimetics, there is muparfostat (PI 88), a d-mannose-based sulfated oligosaccharide mixture, which is used as adjuvant therapeutics for the treatment of hepatocarcinoma [198] and has shown antiviral activity towards poxvirus vaccinia virus (VACV) [199], HSV-1 and HSV-2 [200], DENV, and encephalitic flaviviruses [201]. Muparfostat inhibition of heparanase activity results in reduced viral infection and cell-to-cell transmission [202]. The involvement of heparanase in the virus release might provide a novel route to effective anti-SARS-CoV-2 therapeutics as in the case of another HS mimetics, namely roneparstat (SST0001), already in a clinical trial as an anticancer agent (NCT01764880) [204]. Broad-spectrum antiviral activity has been demonstrated by dispirotripiperazine-based compounds (PDSTPs), small molecules with high binding affinity to HS [203], thus suggesting a potential use to inhibit SARS-CoV-2 entry into host cells. Suramin is the unique broad-spectrum antiviral repurposed drug that has already been shown to inhibit SARS-CoV-2 infection in in vitro cellular tools [206]. This polysulfonated naphthylurea-based small molecule has already been shown to efficiently inhibit infection from HCV [207], HSV-1 [208], Zika virus [209], and others. In Vero E6 cells and primary human airway epithelial cell culture model, suramin inhibited the progression of SARS-CoV-2 infection, possibly preventing the attachment or entry of the virus [206].

In addition to the repurposed drugs listed in Table 3, other approaches that target the HSPG biosynthetic machinery for the treatment of SARS-CoV-2 infection might include the use of synthetic xylosides [9,20,216]. These compounds, composed of a xylose and an aglycone group, compete with HSPG biosynthetic enzymes to bind HS, leading to reduced PG-bound HS and increased xyloside-bound HS. Synthetic xylosides are orally available, easily excreted by the organism, and are advantageous compared with synthetic- or animal-derived HS for potential therapeutic applications because they utilize the host cell biosynthetic apparatus to assemble HS and are thus likely nonimmunogenic [9,216]. To date, the antiviral activity of xylosides remains unexplored. Another group of inhibitors of the HS synthesis is represented by analogs of genistein, a soy-derived isoflavone with structural similarity to 17β-estradiol, which inhibits GAG synthesis by affecting the epidermal growth factor (EGF)-dependent pathway [217]. These compounds have been proven to reduce GAG biosynthesis and derived disorders in human diseases such as mucopolysaccharidoses (MPSs), cancer, and rotavirus infection [218,219,220]. Furthermore, enzymatic methods employing mammalian heparanase and/or sulfatases to remove or to edit the sulfated domains of the HS chains have been explored to interfere with either the attachment of viruses to cell membrane HSPGs or the viral release and cell-to-cell spread [7,99,202,221,222]. Interestingly, heparanase has been shown to affect the bioavailability of signaling molecules such as EGF, Akt, mitogen-activated protein kinase (MAPK)/extracellular signal-regulated kinase (ERK), and vascular endothelial growth factor (VEGF) that are known regulators of viral infections, thus playing an important role in the signaling pathways involved in viral pathogenesis [99]. Studies aimed to explore how heparanase regulates SARS-CoV-2 infection and interacts with the major pro-survival signaling pathways could provide insights to pave the way for novel therapies.

Finally, we recently developed an innovative HSPG-targeting strategy for the cure of some MPS subtypes, inherited human diseases characterized by the accumulation of an excess of the cell surface and extracellular HS leading to the loss of cellular functions, tissue damage, and organ dysfunctions [174]. The strategy, which we called substrate-masking technology, is based on the use of NK1, a natural spliced variant of the hepatocyte growth factor/scatter factor (HGF/SF), which has a high binding affinity for the HS chains [223]. We demonstrated that the recombinant NK1 is able to bind the excess of accumulated HS and to reverse deregulated cellular processes in fibroblasts from MPS-affected patients [174]. NK1 treatment might also be effective against SARS-CoV-2 as well as all the other viruses that require HS binding for attachment and entry into host cells. On the other hand, growth factor receptors are involved in the pathogenesis of many viral infections, and thus they have emerged as potential therapeutic targets against viral diseases [224], including SARS-CoV-2 disease. Indeed, viruses may use growth factor receptors not only to attach to the host cell surface and subsequently internalize into the host cell, but also to target receptor signaling to their replication. To mask HS chains on the host cell surface thus preventing virus attachment, the HGF truncated peptide NK1 could interact with the tyrosine kinase receptor Met, thus affecting the downstream signaling pathways essential for the viral replication. Studies are in progress in our laboratory to evaluate the efficacy of the recombinant NK1 to prevent SARS-CoV-2 infection in vitro, and its molecular mechanism of action.

## 6. Concluding Remarks

The ubiquitously expressed HSPGs play key roles in the pathogenesis of many human viral infections. They not only facilitate viral attachment to the target cells, but may also be involved in the pathways responsible for the internalization, intracellular trafficking, and release of the viral particles. Among the viruses that exploit HSPGs to infect target cells, SARS-CoV-2 has recently raised great public health concern due to its pandemic spread. A consistent amount of data supports the dependence of SARS-CoV-2 on HSPGs for an efficient infection. These data suggest that HSPGs serve as a co-receptor for the viral S protein interaction with the ACE2 entry receptor, thus contributing to viral internalization [66,109,110,129,132,225]. However, as HSPGs have been recognized as key inflammatory mediators in a variety of settings [6,226,227], they could play an important role in the regulation of the host cell immune response to SARS-CoV-2 infection. Thus, it will be intriguing to establish whether and how HSPGs affect the exuberant inflammatory response associated with the severe forms of COVID-19 [228].

It has been widely established that SARS-CoV-2 infection not only involves the respiratory tract but also other organs including the gut, liver, kidney, heart, and pancreas [141,142,143,144,145]. On the other hand, evidence has revealed that the male sex is a risk factor for a more severe disease, including death [172]. In addition, severe disease outcomes have been reported for older people, with young and healthy adults showing a different disease tropism and less severe disease [173]. The precise structural features of HSPGs among the tremendous variety of sulfated HS chains as well as the distinct expression of HSPG synthetic and modifying enzymes within different tissues and organs might contribute to accounting for the differential SARS-CoV-2 tropism, and the distinct susceptibility to infection of different human populations remains an open issue.

Evidence regarding the fundamental roles of the HSPGs in the SARS-CoV-2 pathogenicity has not only provided a better understanding of the virus’s biology and the molecular mechanisms of infection, but has also allowed researchers to identify HSPG-targeted therapies as effective intervention strategies. The potential candidates include HSPG-targeting therapeutics that have already been shown to be effective for the cure of some human diseases including infections from viruses other than SARS-CoV-2. While all of these drugs may represent promising therapeutic options against SARS-CoV-2 infection in humans, specific in vitro studies and in vivo clinical trials are still lacking for most of them. Additional efforts in such a direction might aid the development of antiviral drugs that could be effective for SARS-CoV-2 as well as unforeseeable viruses.

## Figures and Tables

**Table 1:** Viral and host factors involved in the attachment and entry of human viruses.

Virus	Viral Factors	Host Cell Surface Factors	Reference(s)
Coronavirus NL63 (HCoV-NL63)	Envelope glycoprotein (gpE), and membrane protein (M)	Angiotensin-converting enzyme 2 (ACE2) receptor, HSPGs	[26,27,28]
Dengue Virus (DENV)	Capsid protein (C), membrane protein (prM/M) and envelope protein (E)	HSPGs, integrin α3, adhesion molecule-3-grabbing non-integrin (DC-SIGN)	[29,30,31]
Enterovirus A71 (EV-A71)	Capsid VP1-4 proteins	P-selectin glycoprotein ligand-1, annexin II, vimentin, HSPGs, sialylated glycans, fibronectin, prohibitin, cyclophilin A, scavenger receptor class B member 2 (SCARB2)	[32]
Herpes Simplex Virus types 1 and 2 (HSV-1 and -2)	Envelope glycoproteins gB, gC, gD, gE, gG, gH, gI, gJ, gK, gL, gM, and gN	Syndecan-1 and -2, nectin-1, complement component C3b, αvβ6- and αvβ8-integrins	[33,34,35,36]
Human Immunodeficiency Virus (HIV)	Glycoprotein gp120	Trans-activator transcription (Tat) protein, CD4 receptor and coreceptor (e.g., chemokine receptor CCR5 or CXCR4), syndecans	[37,38,39]
Human Hepatitis B Virus (HBV)	Envelope small (S) protein, middle (M) protein, and large (L) protein	Glypican-5, sodium–taurocholate co-transporting polypeptide (NTCP), epidermal growth factor receptor (EGFR), E-cadherin, asiaglycoprotein receptor, transferrin receptor, IL-6 receptor, polymerized human albumin receptor	[40,41,42,43]
Human Hepatitis C Virus (HCV)	Glycoproteins E1 and E2	Apolipoprotein E, syndecan-1 and -4, scavenger receptor class B1 (SR-BI), claudin-1, occludin, T cell immunoglobulin and mucin domain 1(TIM-1)—containing proteins	[44,45,46,47,48]
Human Hepatitis E Virus (HEV)	Open reading frame 2 protein (pORF2)	Asialoglycoprotein receptor ½ (ASGPR1/2), integrin α3, syndecan-1, ATP synthase subunit 5β, glucose-regulated protein 78 (GRP78)	[49,50]
Human Papilloma Virus (HPV)	L1 and L2 proteins	Syndecans, α6 integrin, EGFR, tetraspanins	[51,52,53,54]
Merkel Cell Polyomavirus (MCPyV)	Capsid proteins VP1 and VP2	Sialylated glycans, sulfated HS	[55,56]
Metapneumovirus (HMPV)	Glycoprotein G, small hydrophobic (SH) protein, and fusion (F) protein	Ephrin B2, β1 integrin, HSPGs	[57,58]
Rabies Virus (RABV)	M (matrix) and G (glycoprotein) proteins	Nicotinic acetylcholine receptor (nAChR), neuronal cell adhesion molecule (NCAM), p75 neurotrophin receptor (p75NTR), metabotropic glutamate receptor subtype 2 (mGluR2), HSPGs, phospholipids, gangliosides	[59,60,61]
Respiratory Syncytial Virus (RSV)	Attachment glycoprotein (G), and fusion glycoprotein (F)	Intercellular adhesion molecule-1 (ICAM-1), Toll-like receptor 4 (TLR4), nucleolin, surfactant protein A (SP-A), HSPGs, annexin II	[62,63,64,65]
Severe Acute Respiratory Syndrome Coronavirus-2 (SARS-CoV-2)	Spike (S) protein	ACE2 receptor, neuropilin-1 (NRP1), tyrosine-protein kinase receptor UFO (AXL), HSPGs	[66,67,68,69]

**Table 2:** HS synthetizing and/or modifying enzyme(s) involved in the pathogenesis of human viral infections.

Virus	HS Synthetizing and/or Modifying Enzyme(s)	Role of Enzyme(s) in Viral Pathogenesis	Reference(s)
Cytomegalovirus (CMV)	3-OST	3-*O-*sulfation in HS chains supports viral entry, and cell-to-cell fusion	[100]
Coxsackieviruses B3 variant PD (CVB3 PD)	NDST1 and 6-OST	N- and 6-O-sulfated HS chains mediate viral attachment and internalization	[101]
Dengue Virus (DENV)	Heparanase and cathepsin L	Upregulation of cathepsin L and heparanase by the viral non-structural protein 1 (NS1) binding to HS on endothelial cells triggers syndecan-1 shedding leading to hyperpermeability of endothelial cells in vitro and systemic vascular leakage in vivo	[102,103]
Herpes Simplex Virus type 1 (HSV-1)	3-OST	O-sulfation at C3 position of GlcN residues promotes viral attachment and entry	[33,70,72]
	Heparanase	Upregulation of heparanase in response to viral infection results in the facilitation of the virus spread to uninfected cells and tissues	[104,105]
Human Hepatitis B Virus (HBV)	3-OST	High levels of 3-O-sulfated HS chain suppress viral replication in hepatocytes	[106]
Human Hepatitis C Virus (HCV)	NDST1 and 6-OST	N- and 6-O-sulfation of HS chains are required for viral attachment and infection	[44,47]
Human Hepatitis E Virus (HEV)	6-OST	6-O-sulfation of syndecans is required for viral attachment and infection	[49]
Human Immunodeficiency Virus (HIV)	6-OST	6-O-sulfation of syndecan HS chains is required for gp120 viral protein binding to host cell surface	[37,38]
Human Papilloma Virus serotype 16 (HPV-16)	Sheddases (MMPs and ADAMs) and heparanase	Shedding of syndecan-1 and heparanase processing are essential steps in the viral release from ECM, cellular uptake, and infection	[7,53,107]
Rabies Virus (RABV)	NDST and 6-OST	N- and 6-O-sulfation of HS chains are required for viral attachment and infection	[59]
Respiratory Syncytial Virus (RSV)	NDST	N-sulfation at C-6 position of GlcN triggers viral attachment and cell-membrane fusion	[63,108]
Severe Acute Respiratory Syndrome Coronavirus-2 (SARS-CoV-2)	NDST1 and 6-OST	N- and 6-O-sulfation of HS chains are required for the viral attachment and infection	[66]
	3-OST	3-O-sulfated HSPGs contribute to the viral cell-to-cell fusion	[109]
	Heparanase	Heparanase activity on HSPGs present on the surface of endothelial cells disrupts the endothelial glycocalyx with subsequent loss of endothelial barrier function. Upregulation of heparanase is associated with severe forms of infection	[110]

**Table 3:** HSPG-targeting therapeutics that might be useful against SARS-CoV-2 infection.

Drug	Chemical Type	Mechanism of Action	Reference(s)
Heparin	Glycosaminoglycan	Competes with the binding of the viral S protein to the HS chains of cell surface HSPGs, thus inhibiting entry	[66,81,130,132,134,179,180,181,182,183]
HBPs	Heparin binding peptides conjugated with CMR-197 protein carrier	Inhibit HSPG-dependent viral internalization; escape the endosomal pathway and enter cytoplasm to target cytoplasmic virus; antagonize viral replication and cell-to-cell transmission	[175,184,185,186,187,188,189]
BNTX	7(E)-Benzylidenenaltrexone; opioid receptor antagonist	Disrupts the actin network impairing viral endocytosis	[81]
Lactoferrin	Iron-binding glycoprotein	Prevents the virus internalization by binding HSPGs	[190,191,192,193,194,195,196]
Mitoxantrone	Synthetic anthraquinone derivative	Prevents the viral entry by binding HSPGs	[81,197]
Mupafostat (PI-88)	Highly sulfated, monophosphorylated mannose oligosaccharide	Inhibits heparanase activity, preventing viral release and cell-to-cell spread	[198,199,200,201,202]
PDSTPs	Dispirotripiperazinium derivatives	Bind cell surface HSPGs, inhibiting viral attachment	[203]
Roneparstat (SST0001)	HS mimetics	Prevents the heparanase activity of HS removal from cell surface thus facilitating viral release	[202,204]
Sunitinib	Indolinone derivative; tyrosine kinase receptor inhibitor	Disrupts the actin network impairing viral endocytosis	[81,205]
Suramin	Polysulfonated naphthylurea-based small molecule	Interferes with viral binding and fusion	[206,207,208,209]
Tilorone	2,7-Bis [2-(diethylamino) ethoxy]-9H-fluoren-9-one; amphiphilic cationic molecule	Induces sulfated GAG storage; suppresses viral replication by activating host innate immunity pathways	[81,139,210]
